# Plasma Phosphorylated Tau 217 as a Discriminative Biomarker for Cerebral Amyloid Angiopathy

**DOI:** 10.1111/ene.70066

**Published:** 2025-02-05

**Authors:** Pei‐Feng Hsieh, Hsin‐Hsi Tsai, Chia‐Ju Liu, Bo‐Ching Lee, Ya‐Chin Tsai, Ruoh‐Fang Yen, Jiann‐Shing Jeng, Li‐Kai Tsai

**Affiliations:** ^1^ Department of Neurology National Taiwan University Hospital Taipei Taiwan; ^2^ Department of Neurology National Taiwan University Hospital Hsin‐Chu Branch Hsin‐Chu Taiwan; ^3^ Graduate Institute of Clinical Medicine National Taiwan University College of Medicine Taipei Taiwan; ^4^ Department of Nuclear Medicine National Taiwan University Hospital Taipei Taiwan; ^5^ Department of Medical Imaging National Taiwan University Hospital Taipei Taiwan; ^6^ Department of Nuclear Medicine National Taiwan University Hospital Hsin‐Chu Branch Hsin‐Chu Taiwan

**Keywords:** Alzheimer disease, cerebral amyloid angiopathy, cerebral small vessel disease, phosphorylated tau 217, plasma biomarker

## Abstract

**Background:**

Blood‐based biomarkers may offer a non‐invasive approach to diagnose cerebral amyloid angiopathy (CAA), especially in early‐stage. We evaluated the ability of plasma phosphorylated tau‐217 (p‐tau 217) to differentiate CAA from Alzheimer's disease (AD) and deep perforator arteriopathy (DPA).

**Methods:**

Patients with AD (age 73.7 ± 8.1 years), probable CAA (74.8 ± 6.9 years), or DPA (66.1 ± 10.4 years) were enrolled from memory and stroke clinics at a medical center in Taiwan. All participants received amyloid and tau PET scans. Plasma biomarkers were measured via a SIMOA immunoassay platform. The diagnostic utility of p‐tau 217 was assessed using ROC analyses and the Youden cutoff. Associations between plasma p‐tau 217 and neuroimaging variables in CAA were explored.

**Results:**

Patients with CAA had lower plasma p‐tau 217 (0.69 ± 0.76 vs. 1.28 ± 0.97 pg/mL, *p* < 0.001) and a lower p‐tau 217/Aβ40 ratio (0.003 ± 0.002 vs. 0.006 ± 0.003, *p* < 0.001) than the AD group but higher levels than the DPA group (p‐tau 217, 0.27 ± 0.13 pg/mL, *p* = 0.001; p‐tau 217/Aβ40, 0.001 ± 0.0005, *p* < 0.001), although adjustment attenuated the difference in p‐tau 217 between CAA and DPA. Plasma Aβ40, Aβ42, and Aβ40/Aβ42 were not significantly different between groups. Plasma p‐tau 217 had moderate to good diagnostic utility to differentiate CAA vs. AD (sensitivity, 64.4%; specificity, 89.5%; AUC, 0.809) and CAA vs. DPA (sensitivity, 67.8%; specificity, 100%; AUC, 0.855). In CAA, p‐tau 217 significantly correlated with the severity of CAA, amyloid PET signal intensity, and lobar microbleed count (*p* < 0.001).

**Conclusions:**

Plasma p‐tau 217 may represent a non‐invasive biomarker for distinguishing cerebral amyloid angiopathy (CAA) from other conditions, including AD and DPA.

## 1 Introduction

Cerebral amyloid angiopathy (CAA), characterized by the progressive deposition of β‐amyloid plaques (Aβ) within the walls of cortical and leptomeningeal small arterioles, is diagnosed using the Boston criteria V2.0 [1]. Due to the invasiveness and low feasibility of brain biopsy, the diagnosis of CAA relies on neuroradiological detection of multiple strictly lobar hemorrhagic lesions, including intracerebral hemorrhage (ICH), cerebral microbleeds (CMB), and cortical superficial siderosis (cSS) [1]. The CAA‐related structural lesions observed on MRI essentially represent vascular‐mediated brain damage, rather than CAA‐laden vessels [2]. These MRI markers only provide indirect evidence of late consequences, and the detection of early‐stage CAA remains difficult [3].

In contrast to the parenchymal Aβ deposition in Alzheimer's disease (AD), which mostly involves the 42‐amino acid form (Aβ42), the deposits in CAA contain both Aβ40 and Aβ42, with Aβ40 being the major isoform [4]. There is growing interest in the identification of CSF biomarkers that could facilitate early detection of sporadic CAA [4]. Although evidence indicates that patients with CAA have lower levels of Aβ40 in CSF compared to patients with AD or healthy controls, the invasive nature of CSF sampling limits its clinical applicability [4, 5].

In the context of increased use of anti‐amyloid therapies, distinguishing between coexisting CAA and AD has become crucial due to the increased risk of amyloid‐related imaging abnormalities (ARIA) [6]. Additionally, distinguishing between CAA and deep perforator arteriopathy (DPA) in patients with spontaneous lobar ICH is vital for providing appropriate care and mitigating the risk of recurrent bleeding.

The levels of phosphorylated tau proteins (p‐tau 181, p‐tau 217, and p‐tau 231) indicate the amyloid plaque burden in AD; thus, these proteins may have utility for the diagnosis of CAA [7]. Recent findings suggested plasma p‐tau 217 is a promising biomarker for differentiating AD from healthy controls and is more effective than other forms of p‐tau [8]. To our knowledge, no studies have investigated whether plasma p‐tau 217 is elevated in CAA or compared this marker in CAA and AD.

To identify non‐invasive, cost‐effective alternatives to CSF sampling and PET imaging, we explored the application of plasma biomarkers, in particular p‐tau 217, for distinguishing CAA from AD and DPA (as a non‐amyloid comparison). As a secondary objective, we assessed the correlations between plasma biomarkers and neuroimaging abnormalities in CAA to delineate the underlying pathological changes.

## 2 Methods

### 2.1 Ethical Approval and Patient Consent

This study was approved by the Institutional Review Board of the Ethics Committee of National Taiwan University Hospital (201903069RINB). Informed consent was obtained from all patients and/or their relatives.

### 2.2 Study Subjects

We prospectively included patients between September 2018 and October 2023 at National Taiwan University Hospital and National Taiwan University Hospital Bei‐Hu Branch. Patients clinically diagnosed with vascular cognitive impairment or with a history of symptomatic spontaneous ICH who met the criteria for probable CAA based on the modified Boston criteria 1.5 (up to May 2022) or 2.0 criteria (June 2022 onwards) [1, 9] were enrolled from memory clinics or stroke clinics. We retrospectively confirmed all enrolled subjects met the diagnostic standard for probable CAA based on the Boston criteria V2.0 [1]. The CAA‐related small vessel disease (CAA‐SVD) score was derived based on imaging features [10].

As comparison groups, we enrolled patients with probable AD who fulfilled the diagnostic criteria according to the National Institute on Aging and Alzheimer's Association (NIA‐AA) [11]. Patients who had survived symptomatic ICH with evidence of deep bleeds, such as hematomas or microbleeds, and no identified macrovascular causes of ICH were classified as having deep perforator arteriopathy (DPA) and were included as a non‐amyloid SVD comparison group [12, 13].

**FIGURE 1:**
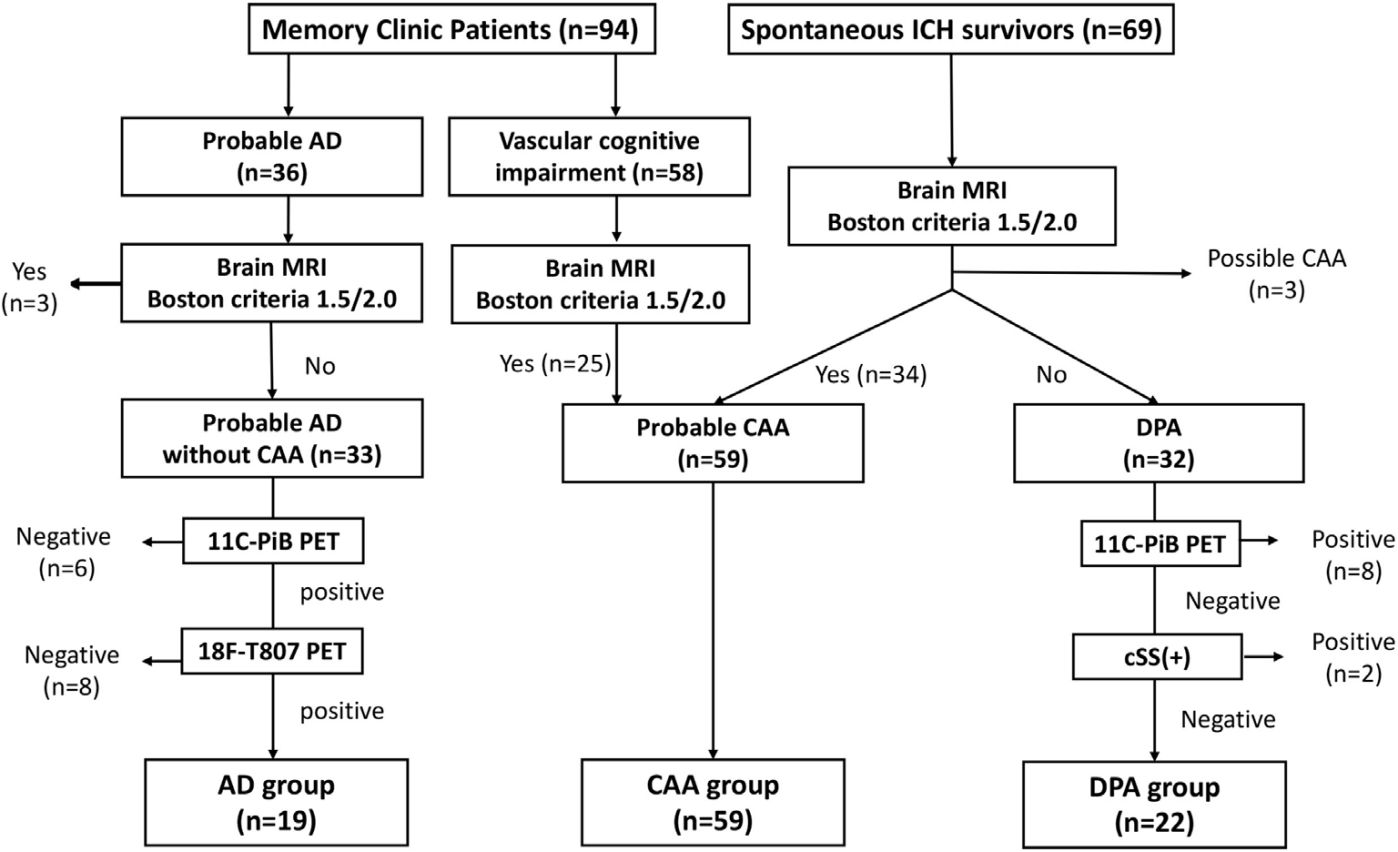
Flowchart of enrollment in this study. Fifty‐nine probable cases of CAA were identified (25 patients with cognitive impairment and 34 survivors of ICH). Additionally, 19 patients with AD and 22 patients with DPA were included as comparison groups.

Eligible patients for whom neuroimaging studies (including MRI, amyloid PET, tau PET) and plasma biomarker measurements were completed were included. As shown in the enrollment flowchart (Figure 1), patients with AD who had either a negative amyloid scan (*n* = 6), a negative tau scan (*n* = 8), or who met the diagnostic standard for probable CAA per the Boston criteria V2.0 (*n* = 3) were excluded; patients with DPA who had a positive amyloid scan (*n* = 8) or cSS (*n* = 2) were also excluded due to the potential for coexisting AD or CAA.

### 2.3 Brain MRI Acquisition and Analysis

Brain MRIs were acquired using a 3‐Tesla scanner (Siemens Verio, TIM or mMR, Siemens Medical Solutions, Malvern, PA, USA). The imaging protocol included T1‐weighted magnetization‐prepared rapid gradient‐echo imaging (MPRAGE, flip angle 9, repetition time/echo time = 1460/2.39 ms, field of view = 25.6 cm, slice thickness = 1 mm), T2‐weighted imaging (repetition time/echo time = 3530/83 ms, field of view = 23 cm, slice thickness = 5 mm), FLAIR (repetition time/echo time = 10,000/89 ms, field of view = 23 cm, slice thickness = 5 mm), susceptibility‐weighted imaging (SWI, flip angle 15, repetition time/echo time = 28/20 ms, matrix number = 221 × 320, field of view = 23 cm, slice thickness = 2 mm), as previously described [14].

MRI markers were evaluated based on the Standards for Reporting Vascular Changes on Neuroimaging criteria 2.0 (STRIVE2) [15]. The number of CMBs and the presence of cSS were evaluated using axial SWI sequences [16, 17]. CMBs in the lobar and deep regions were counted. Lacunes were evaluated in the supratentorial region and defined as “round or ovoid, subcortical, fluid‐filled cavities, from 3 to 15 mm in diameter.” The volume of white matter hyperintense (WMH) lesions was calculated using an in‐house semiautomated algorithm [18, 19]. After registering the T2 FLAIR volume to the T1‐weighted volume, a minimum intensity threshold of 1.5 SDs was applied. The volume was extracted from the ICH‐free hemisphere and multiplied by two.

Perivascular spaces (PVS) were evaluated on T2‐weighted imaging and defined as sharply delineated structures measuring < 3 mm following the course of perforating or medullary vessels [20]. Severity of enlarged PVS at the levels of the basal ganglia and centrum semiovale was assessed on the more affected side of the brain using a four‐point scale: grade 0 (no enlarged PVS), grade 1 (1–10), grade 2 (11–20), grade 3 (21–40), and grade 4 (> 40) [21].

MRI scans were also processed using FreeSurfer software v6.0.0 (http://surfer.nmr.mgh.harvard.edu/) to extract the volume of the hippocampus from the T1‐weighted MPRAGE sequence.

### 2.4 PET Acquisition and Analysis

Radiotracers were prepared at the Cyclotron and Radiopharmaceutical Laboratory of NTUH. PET images (Discovery ST, GE Healthcare) were acquired over 30 min, starting 40 min after the injection of 10 mCi ^11^C‐Pittsburgh compound B (PiB) or ^18^F‐T807. Using a previously described protocol [14], PET data were reconstructed via ordered set expectation maximization and corrected for attenuation.

Amyloid scan positivity was determined by two nuclear medicine specialists (authors C.‐J. L. and Y.‐C T.) using a previously proposed approach for visual assessment [22]. Any discordant results were resolved by consensus. The high‐resolution T1‐weighted MRI was auto‐segmented using MNI space/coordinates based on the AAL maximum probability atlas with PMOD software (PNEURO module). The PET data were matched and normalized to the corresponding T1 image. Standardized uptake values (SUV) were calculated for the cerebellar cortex and cortical ribbon regions of interest (ROIs).

For T807 scans, a meta‐temporal ROI that included the entorhinal cortex, amygdala, parahippocampal gyrus, fusiform gyrus, and inferior and middle temporal gyri was created as an AD signature [23, 24]. A cutoff of SUVR 1.26 (reference: cerebellar cortex) was applied to determine tau PET positivity based on the results for previous cohorts using the mean and 2 standard deviations (SD) of T807 uptake from amyloid‐negative and cognitively unimpaired participants [25].

### 2.5 Plasma Biomarker Analysis

Venous blood (10 mL) was drawn from each participant at enrolment and centrifuged (2500 *g* for 15 min) within 3 h. DNA was isolated, and the *APOE* epsilon alleles (e2, e3, and e4) were genotyped by PCR. The plasma was aliquoted and stored at −80°C. Plasma levels of total tau, amyloid‐42, and amyloid‐40 were measured using the Simoa immunoassay platform (Quanterix, Billerica, MA, USA) [26]. Plasma levels of phosphorylated tau‐217 were measured using the commercial ALZpath pTau217 assay [27]. All measurements were performed in duplicate by blinded, board‐certified technicians, and average concentrations were calculated.

### 2.6 Statistical Analysis

Statistical analysis was performed using SPSS version 19.0. Continuous data are presented as mean ± SD or median (25th–75th percentile), and categorical data as percentages (%). Fisher's exact test or Pearson's *χ* ^2^ test were used to compare categorical variables, as appropriate, and the Mann–Whitney *U*‐test was used for continuous variables. Quantile regression adjusted for confounding variables, and linear regression was performed to evaluate the correlations between p‐tau 217 and other variables. The false discovery rate (FDR) method was applied to correct for multiple comparisons of different plasma biomarkers. Diagnostic value was assessed via receiver operating characteristic (ROC) analysis. We determined cut‐off values using the Youden index and calculated the area under the curve (AUC), sensitivity, and specificity. Delong's test was used to compare the AUCs of different biomarkers, and delta AUC was calculated using bootstrap methods. A two‐tailed *p* < 0.05 was considered significant.

## 3 Results

The final analyses included 59 patients in the probable CAA group (mean age 74.8 ± 6.9, 46% male) and 19 patients in the AD group (mean age 73.7 ± 8.1, 32% male; Figure 1). The demographic features, clinical variables, and imaging biomarkers of the CAA and AD groups are compared in Table 1. The patients with CAA had similar age and sex distributions as the patients with AD; however, the CAA group exhibited better cognitive performance with higher mini‐mental state examination (MMSE) scores [28] (21.3 ± 7.8 vs. 17.2 ± 6.3, *p* = 0.018) and lower clinical dementia rating (CDR) scores [29] (0.5 [0.5–1] vs. 1.0 [0.5–1.5], *p* = 0.022). As expected, the AD group had lower burdens of SVD imaging biomarkers, including fewer lobar microbleeds (0 [0–0] vs. 4 [2–12.5], *p* < 0.001) and lower WMH (3.3 [2.0–6.0] vs. 9.1 [4.8–15.0] mL, *p* = 0.002).

**TABLE 1:** Comparison of the demographic and radiological characteristics of the patients with CAA and AD or DPA.

	CAA (*n* = 59)	AD (*n* = 19)	*p*	DPA (*n* = 22)	*p*
Age, years	74.8 ± 6.9	73.7 ± 8.1	0.662	66.1 ± 10.4	< 0.001*
Male sex, %	27 (46%)	6 (32%)	0.276	17 (77%)	0.011*
eGFR, mL/min/1.73 m^2^	76.9 ± 28.8	79.6 ± 16.9	0.845	73.8 ± 23.5	0.755
MMSE	21.3 ± 7.8	17.2 ± 6.3	0.018	22.6 ± 8.6	0.368
CDR	0.5 (0.5–0.5)	1 (0.5–1.5)	0.022	0.5 (0–0.5)	0.087
*ApoE4* frequency, %	21 (38%)	6 (32%)	0.642	3 (14%)	0.040*
CAA‐SVD score	2 (2–3.5)	1 (0–1)	< 0.001*	2 (1–3)	0.288
Number of lobar microbleeds	4 (2–12.5)	0 (0–0)	< 0.001*	4.5 (2–17)	0.617
Prevalence of lobar microbleeds (%)	57 (97%)	2 (11%)	< 0.001*	19 (86%)	0.088
Number of deep microbleeds	0 (0–0)	0 (0–0)	0.002*	3.5 (0.25–8)	< 0.001*
WMH volume, mL	9.1 (4.8–15.0)	3.3 (2.0–6.0)	0.002*	10.6 (6.4–16.7)	0.775
cSS(+) rate, %	21 (36%)	0 (0%)	0.002*	0 (0%)	0.001*
Total cSS score	0 (0–1)	0 (0–0)	0.003*	0 (0–0)	0.001*
CSO‐PVS grade	2 (2–3)	3 (2–3)	0.713	2 (1–2.75)	0.067
BG‐PVS grade	2 (1–3)	2 (1–2)	0.365	3 (2–3)	0.015*
Total lacune count	0 (0–1)	0 (0–0)	0.296	1.5 (0–3)	< 0.001*
Hippocampus, mm^3^	3452.2 ± 710.1	2914.4 ± 454.5	< 0.001*	3588.1 ± 509.3	0.139

*Note:* Values are mean (± standard deviation), median (interquartile range), or number (percentage). *p‐value < 0.05. The *p‐*values next to the AD column indicate the comparison between the CAA and AD groups; *p*‐values next to the DPA column indicate the comparison between the CAA and DPA groups.

Abbreviations: AD, Alzheimer's disease; BG, basal ganglia; CAA, cerebral amyloid angiopathy; CDR, Clinical Dementia Rating; CSO, central semiovale; cSS, cortical superficial siderosis; DPA, deep perforator arteriopathy; eGFR, estimated glomerular filtration rate; PVS, perivascular space; MMSE, Mini‐Mental Status Exam; WMH, white matter hyperintensities.

Twenty‐two patients were enrolled in the DPA group (mean age 66.1 ± 10.4, 77% male) as a non‐amyloid SVD comparison (Table 1). The DPA group was younger (*p* < 0.001), contained a higher proportion of males (77% vs. 46%, *p* = 0.011), and had a lower frequency of the *ApoE4* allele (14% vs. 38%, *p* = 0.040) than the CAA group. As expected, patients with DPA had more deep microbleeds (3.5 [0.25–8] vs. 0 [0–0], *p* < 0.001), more severe BG‐PVS (3 [2, 3] vs. 2 [1–3], *p* = 0.015), and more lacunes (1.5 [0–3] vs. 0 [0–1], *p* < 0.001).

### 3.1 Comparison of the Ability of Plasma Biomarkers to Differentiate Between CAA and AD

**FIGURE 2:**
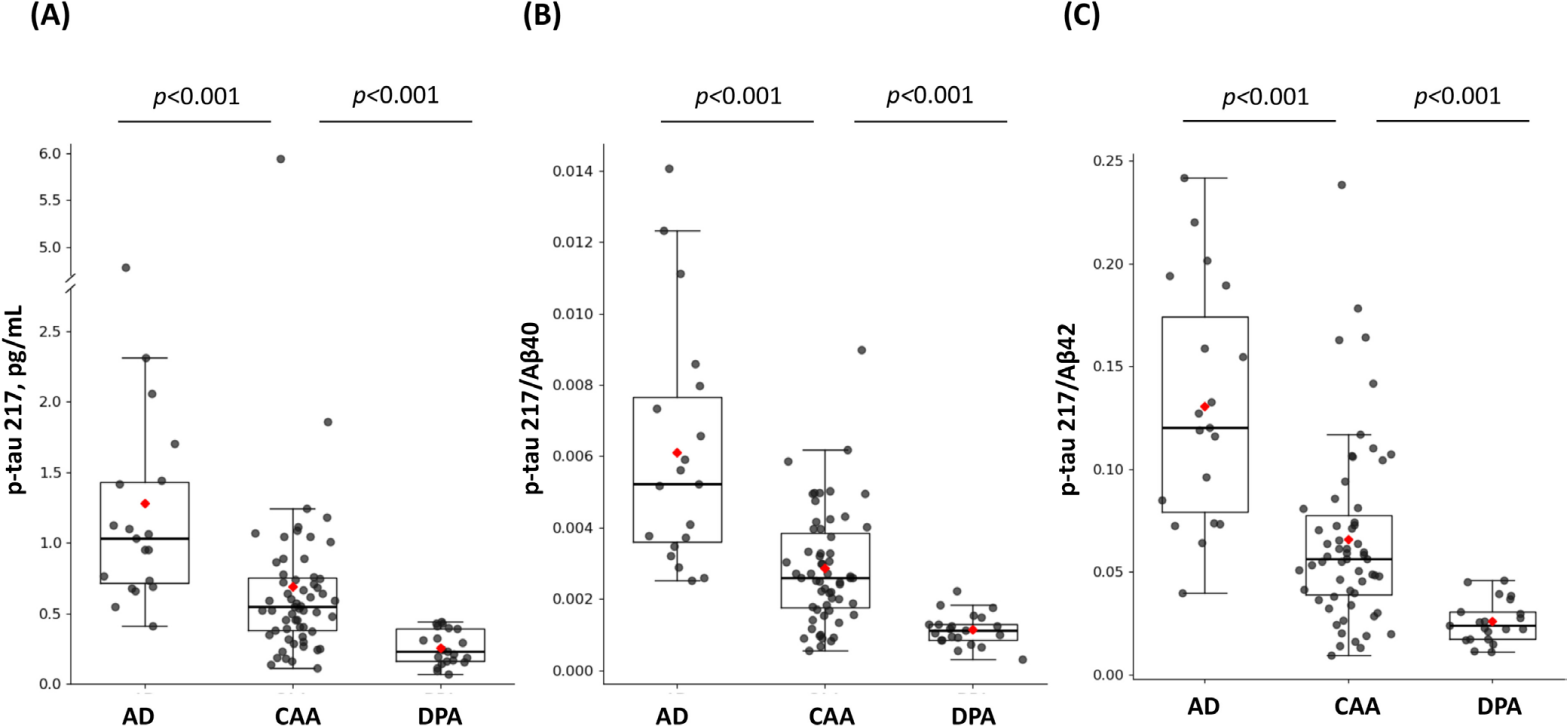
Levels of plasma biomarkers in AD, CAA, and DPA. Box plots and scatter plots of (A) p‐tau 217, (B) p‐tau 217/Aβ40, and (C) p‐tau 217/Aβ42 in the AD, CAA, and DPA groups. Red diamonds represent the means. The boxes encompass 50% of the samples, and the whiskers extend 1.5 quartiles to each side of the box. Significant differences are indicated, including *p*‐values.

Patients with CAA had lower levels of p‐tau 217 than the AD group (0.69 ± 0.76 pg/mL vs. 1.28 ± 0.97 pg/mL, *p* < 0.001, Figure 2A). In terms of composite biomarkers, CAA patients also had a lower p‐tau 217/t‐tau ratio (0.29 ± 0.22 vs. 0.51 ± 0.37, *p* = 0.013), lower p‐tau 217/Aβ40 ratio (0.003 ± 0.002 vs. 0.006 ± 0.003, *p* < 0.001, Figure 2B), and lower p‐tau 217/Aβ42 ratio (0.066 ± 0.044 vs. 0.131 ± 0.058, *p* < 0.001, Figure 2C) than the AD group. The differences in p‐tau 217, p‐tau 217/Aβ40, and p‐tau 217/Aβ42 remained significant after adjustment (all *p* < 0.05; Table 2). Plasma Aβ40, Aβ42, and the Aβ42/40 ratio were not significantly different between the CAA and AD groups (all *p* > 0.05).

**TABLE 2:** Comparison of core plasma biomarker levels in CAA, AD, and DPA.

	CAA (*n* = 59)	AD (*n* = 19)	*p*	Adjusted, *p* ^a^	FDR‐corrected *p* ^b^	DPA (*n* = 22)	*p*	Adjusted, *p* ^a^	FDR‐corrected *p* ^b^
Aβ 40, pg/mL	232.05 ± 87.60	205.57 ± 50.99	0.202	0.386	0.494	229.19 ± 73.42	0.962	0.027*	0.064
Aβ 42, pg/mL	10.77 ± 5.07	9.59 ± 3.61	0.399	0.432	0.494	10.82 ± 5.33	0.298	0.684	0.754
p‐tau 217, pg/mL	0.69 ± 0.76	1.28 ± 0.97	< 0.001*	< 0.001*	0.001*	0.27 ± 0.13	< 0.001*	0.032*	0.064
t‐tau, pg/mL	2.92 ± 2.30	3.21 ± 1.72	0.190	0.023*	0.046*	2.69 ± 2.11	0.168	0.689	0.754
Aβ 42/40	0.05 ± 0.01	0.05 ± 0.01	0.811	0.684	0.684	0.05 ± 0.02	0.628	0.754	0.754
p‐tau217/t‐tau	0.29 ± 0.22	0.51 ± 0.37	0.013*	0.061	0.098	0.14 ± 0.08	0.040*	0.074	0.118
p‐tau217/Aβ40	0.003 ± 0.002	0.006 ± 0.003	< 0.001*	< 0.001*	< 0.001*	0.001 ± 0.0005	< 0.001*	0.001*	0.003*
p‐tau217/Aβ42	0.066 ± 0.044	0.131 ± 0.058	< 0.001*	< 0.001*	< 0.001*	0.026 ± 0.010	< 0.001*	0.001*	0.002*

*Note:* Values are mean (± standard deviation).

Abbreviations: Aβ, amyloid beta; AD, Alzheimer's disease; CAA, cerebral amyloid angiopathy; DPA, deep perforator arteriopathy; eGFR, estimated glomerular filtration rate; FDR, false discovery rate; p‐tau, phosphorylated tau; t‐tau, total tau.

^a^ Adjusted for age by quantile regression.

^b^ Adjusted for age by quantile regression and false discovery rate correction.

* *p*‐value < 0.05.

To address the risk of potential concomitant AD pathology in CAA, we performed a sensitivity analysis of the CAA patients with negative tau PET scans (*n* = 36, 61% of the CAA group). Similarly to the findings in the whole CAA group, the tau(‐) CAA subgroup had significantly lower p‐tau 217, p‐tau 217/t‐tau, p‐tau 217/Aβ40, and p‐tau 217/Aβ42 compared to the AD group (all *p* < 0.05) and comparable plasma Aβ40, Aβ42, and Aβ42/40 ratio (Table S1).

**FIGURE 3:**
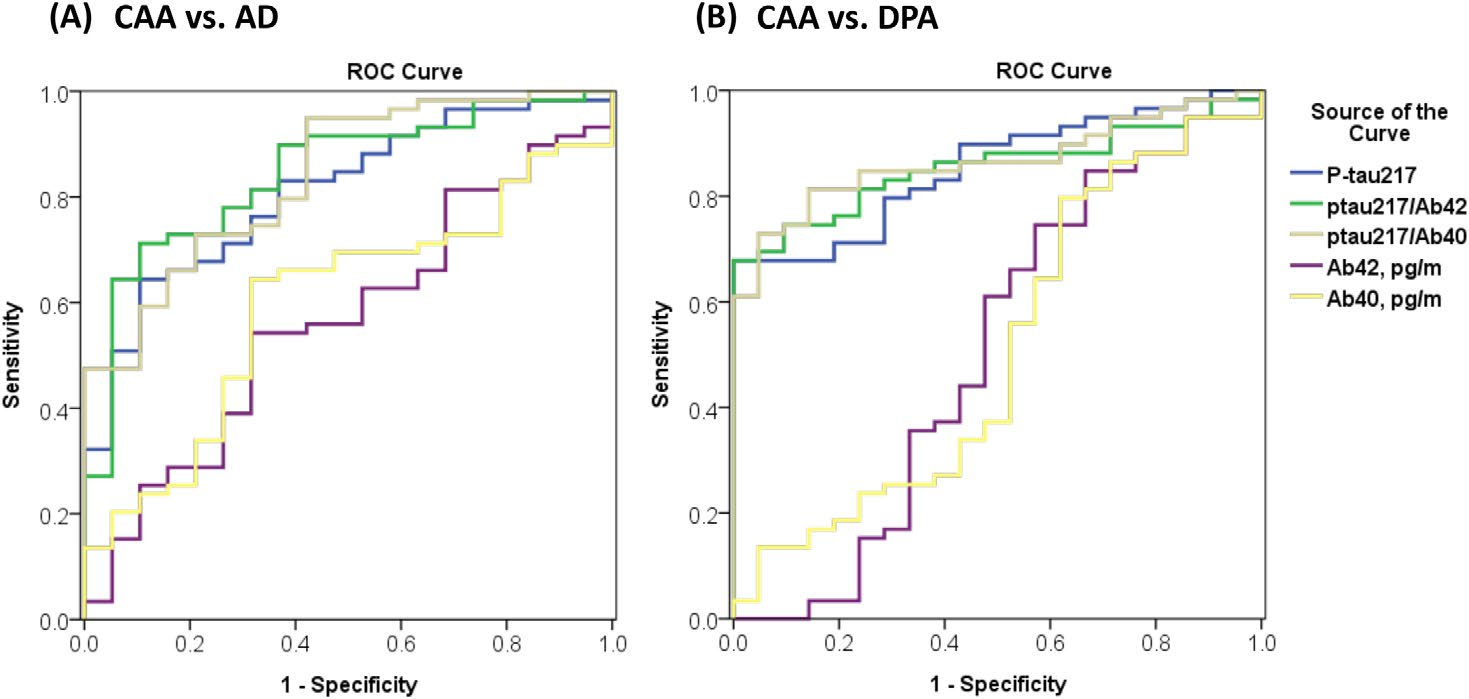
Diagnostic performance of the plasma biomarkers. Receiver‐operating characteristic curves (ROC) of Aβ40, Aβ42, p‐tau217, p‐tau 217/Aβ40, and p‐tau 217/Aβ42 for differentiation of (A) CAA vs. AD and (B) CAA vs. DPA. Compared to plasma beta amyloid alone, p‐tau 217 and the composite biomarkers (p‐tau 217/Aβ40 and p‐tau 217/Aβ42) had much larger areas under the curve (AUC) for CAA vs. AD and CAA vs. DPA.

The diagnostic ability of plasma biomarkers to differentiate CAA and AD is shown in Table 3. Of all the biomarkers tested (Figure 3), p‐tau 217 exhibited diagnostic superiority for differentiating CAA from AD, with an AUC of 0.809 (95% CI, 0.707–0.912); p‐tau 217 significantly outperformed Aβ40, Aβ42, the Aβ42/40 ratio, and total tau (all *p* < 0.05). Using the cutoff of < 0.650 pg/mL indicated by the Youden index, plasma p‐tau 217 provided a sensitivity of 64.4% (95% CI 50.9%–76.5%) and specificity of 89.5% (95% CI 66.9%–98.7%) for differentiation of CAA from AD. Some composite biomarkers, especially the p‐tau 217/Aβ40 and p‐tau 217/Aβ42 ratios, provided slightly higher AUC values (p‐tau 217/Aβ40: 0.839, p‐tau 217/Aβ42:0.844); however, the diagnostic utility of these composite markers was not significantly better than p‐tau 217 alone (both *p* > 0.05).

**TABLE 3:** Diagnostic performance of core plasma biomarkers for differentiation of CAA vs. AD and CAA vs. DPA.

	Cutoff	Sensitivity	Specificity	AUC	△AUC	*p*
*CAA vs. AD*						
Aβ40, pg/mL	> 195.710	0.644 (0.509–0.765)	0.684 (0.435–0.874)	0.598 (0.459–0.736)	0.211 (0.008–0.414)	0.041
Aβ42, pg/mL	> 9.305	0.542 (0.408–0.673)	0.684 (0.435–0.874)	0.565 (0.421–0.708)	0.244 (0.057–0.431)	0.010
p‐tau 217, pg/mL	< 0.650	0.644 (0.509–0.765)	0.895 (0.669–0.987)	0.809 (0.707–0.912)	—	—
Tau, pg/mL	< 3.073	0.729 (0.597–0.836)	0.526 (0.289–0.756)	0.600 (0.453–0.748)	0.209 (0.040–0.377)	0.015
Aβ42/40	> 0.043	0.644 (0.509–0.765)	0.526 (0.289–0.756)	0.518 (0.354–0.682)	0.290 (0. 091–0.491)	0.004
p‐tau 217/t‐tau	< 0.258	0.593 (0.458–0.719)	0.737 (0.488–0.909)	0.690 (0.552–0.829)	0.1 (−0.006 to 0.247)	0.063
p‐tau 217/Aβ40	< 0.0051	0.949 (0.859–0.989)	0.579 (0.335–0.798)	0.839 (0.743–0.936)	−0.030 (−0.095 to 0.034)	0.359
p‐tau 217/Aβ42	< 0.073	0.712 (0.579–0.822)	0.895 (0.669‐0.987)	0.844 (0.747–0.940)	−0.035 (−0.098 to 0.028)	0.279
*CAA vs. DPA*						
Aβ40, pg/mL	> 173.28	0.796 (0.672–0.890)	0.381 (0.181–0.616)	0.508 (0.354–0.663)	0.346 (0.204–0.489)	< 0.001
Aβ42, pg/mL	< 14.106	0.847 (0.730–0.928)	0.333 (0.146–0.570)	0.506 (0.341–0.671)	0.349 (0.133–0.565)	0.002
p‐tau 217, pg/mL	> 0.448	0.678 (0.544–0.794)	1.0 (0.839–1.000)	0.855 (0.774–0.935)	—	—
Tau, pg/mL	> 2.775	0.424 (0.296–0.559)	0.762 (0.528–0.918)	0.562 (0.414–0.710)	0.293 (0.151–0.434)	< 0.001
Aβ42/40	< 0.058	0.881 (0.771–0.951)	0.381 (0.181–0.616)	0.531 (0.364–0.699)	0.323 (0.130–0.517)	0.001
p‐tau 217/t‐tau	> 0.188	0.627 (0.492–0.750)	0.857 (0.636–0.969)	0.756 (0.647–0.866)	0.098 (−0.013 to 0.210)	0.084
p‐tau 217/Aβ40	> 0.046	0.746 (0.616–0.850)	0.905 (0.696–0.989)	0.856 (0.775–0.936)	−0.001 (−0.067 to 0.065)	0.981
p‐tau 217/Ab42	> 0.0018	0.729 (0.597–0.836)	0.952 (0.762–0.999)	0.867 (0.790–0.944)	−0.012 (−0.066 to 0.042)	0.660

*Note:* *p*‐values are the results of Delong's test to compare the AUCs of other plasma biomarkers vs. the AUC of p‐tau 217.

Abbreviations: Aβ, amyloid beta; AD, Alzheimer's disease; AUC, area under the curve; CAA, cerebral amyloid angiopathy; DPA, deep perforator arteriopathy; p‐tau, phosphorylated tau; t‐tau, total tau.

### 3.2 Comparison of the Ability of Plasma Biomarkers to Differentiate Between CAA and DPA

The levels of the principal plasma biomarkers in the patients with DPA are presented in Table 2. Compared to the DPA group, the CAA group had higher p‐tau 217 (0.69 ± 0.76 vs. 0.27 ± 0.13 pg/mL, *p* < 0.001, Figure 2A), a higher p‐tau 217/t‐tau ratio (0.29 ± 0.22 vs. 0.14 ± 0.08, *p* = 0.040), a higher p‐tau 217/Aβ40 ratio (0.003 ± 0.002 vs. 0.001 ± 0.0005, *p* < 0.001, Figure 2B), and a higher p‐tau 217/Aβ42 ratio (0.066 ± 0.044 vs. 0.026 ± 0.010, *p* < 0.001, Figure 2C); the significance of the difference in p‐tau 217 was attenuated by adjustment (*p* = 0.064).

As shown in Table 3, most biomarkers had limited diagnostic utility to differentiate CAA from DPA. However, using a cutoff of > 0.448 pg/mL, plasma p‐tau 217 provided a sensitivity of 67.8% (95% CI, 54.4%–79.4%) and a specificity of 100% (95% CI, 83.9%–100.0%). The AUC of p‐tau 217 (0.855; 95% CI, 0.774–0.935) was significantly higher than the AUCs of Aβ40, Aβ42, the Aβ42/40 ratio, and total tau (Figure 3). Composite biomarkers, including p‐tau 217/Aβ40 (AUC: 0.856) and p‐tau 217/Aβ42 (AUC: 0.867) had similar diagnostic utility as p‐tau 217 alone.

Exploratory analysis comparing the plasma p‐tau 217 levels in patients with DPA, mild CAA (CAA‐SVD score ≤ 2), and severe CAA (CAA‐SVD score > 3) showed that the p‐tau 217 level was significantly higher in severe CAA compared to mild CAA (0.93 ± 1.15 vs. 0.47 ± 0.24, *p* = 0.0001) and was also significantly elevated in mild CAA compared to DPA (0.47 ± 0.24 vs. 0.27 ± 0.13, *p* = 0.003).

### 3.3 Subgroup Analyses of Plasma Biomarkers in Patients With Cognitive Impairment and Patients With ICH

To explore the diagnostic utility of plasma p‐tau 217 in different clinical scenarios, we performed separate subgroup analyses of patients with cognitive impairment (CI) and patients with ICH. Aβ and Tau plasma biomarkers were not significantly different between the CAA‐CI and CAA‐ICH groups (Table S2).

In patients with cognitive impairment, including AD (*n* = 19) and CAA‐cognitive impairment (*n* = 25), the optimal cutoff for p‐tau 217 provided a sensitivity of 64.0% (95% CI, 42.5%–82.0%), specificity of 89.5% (95% CI, 66.8%–98.7%), and AUC of 0.787 (95% CI, 0.655–0.920) for differentiating CAA from AD (Table S3).

In survivors of ICH, including CAA‐related ICH (*n* = 34) and DPA (*n* = 22), the optimal cutoff for p‐tau 217 provided a sensitivity of 67.6% (95% CI, 49.5%–82.6%), specificity of 95.5% (95% CI, 77.2%–99.9%), and AUC of 0.850 (95% CI, 0.752–0.948) for differentiating CAA from DPA (Table S3).

### 3.4 Correlations Between Plasma p‐Tau 217 and Neuroimaging Markers in CAA

To investigate the mechanisms underlying elevated plasma p‐tau 217 in CAA, we explored the correlations between imaging biomarkers and p‐tau 217 levels in the CAA group (Table 4). Plasma p‐tau 217 levels significantly correlated with the number of lobar microbleeds (*β* = 0.408, 95% CI 0.166–0.650, *p* = 0.001) and CAA‐SVD score (*β* = 0.298, 95% CI 0.045–0.551, *p* = 0.022). After adjusting for age, eGFR, and *ApoE4* status, significant correlations remained between plasma p‐tau 217 and lobar microbleeds (*β* = 0.412, 95% CI 0.166–0.657, *p* = 0.001), CAA‐SVD score (*β* = 0.366, 95% CI 0.125–0.607, *p* = 0.004), and amyloid load on PET (*β* = 0.301, 95% CI 0.033–0.569, *p* = 0.028). However, no significant correlation was observed between plasma p‐tau217 levels and cerebral tau load (*p* = 0.317).

**TABLE 4:** Correlations between plasma p‐tau 217 and imaging markers in CAA.

	Univariate		Multivariate^a^	
	*β* (95% CI)	*p*	*β* (95% CI)	*p* ^a^
Age	0.032 (−0.233, 0.297)	0.809	−0.124 (−0.394, 0.147)	0.364
Meta‐temporal T807 SUVR	0.059 (−0.205, 0.324)	0.655	0.139 (−0.137, 0.417)	0.317
Whole cortex PiB SUVR	0.180 (−0.081, 0.441)	0.172	0.301 (0.033, 0.569)	0.028*
Number of lobar CMBs	0.408 (0.166, 0.650)	0.001*	0.412 (0.166, 0.657)	0.001*
Total cSS score	0.096 (−0.168, 0.359)	0.472	0.267 (−0.019, 0.555)	0.067
WMH volume	0.097 (−0.166, 0.361)	0.463	0.146 (−0.157, 0.450)	0.339
CSO‐EPVS grade	0.198 (−0.061, 0.459)	0.131	0.214 (−0.048, 0.476)	0.107
CAA‐SVD score	0.298 (0.045, 0.551)	0.022*	0.366 (0.125, 0.607)	0.004*

*Note:* Values are beta coefficients calculated by linear regression with univariate analysis in the second column.

Abbreviations: CAA, cerebral amyloid angiopathy; CMB, cerebral microbleeds; CSO, central semiovale; cSS, cortical superficial siderosis; eGFR, estimated glomerular filtration rate; EPVS, enlarged perivascular space; SVD, small vessel disease; SUVR, standardized uptake value ratio; WMH, white matter hyperintensities.

* *p*‐value < 0.05.

^a^ Multivariate model was adjusted for age, eGFR, and *ApoE4* status.

## 4 Discussion

We compared plasma biomarkers, including Aβ40, Aβ42, t‐tau, and p‐tau 217, in CAA, AD, and DPA. Patients with CAA had lower p‐tau 217 levels than patients with AD but higher levels than patients with DPA. Moreover, plasma p‐tau 217, p‐tau 217/Aβ40, and the p‐tau 217/Aβ42 ratio effectively discriminated between CAA and AD and between CAA and DPA; Aβ40, Aβ42, and the Aβ40/Aβ42 ratio did not. In probable CAA, plasma p‐tau 217 levels correlated positively with the severity of CAA, particularly lobar microbleeds and the cerebral amyloid burden, but not the tau burden.

Our findings indicate plasma p‐tau 217 has potential as a non‐invasive biomarker to differentiate CAA from AD or DPA pathology. The CAA group had significantly lower plasma p‐tau 217 levels than the AD group, consistent with recent AD research that recognized soluble p‐tau as an indicator of the parenchymal amyloid burden and its role in driving the development of insoluble tau aggregates [30]. Interestingly, our CAA group also had higher plasma p‐tau 217 than the non‐amyloid DPA controls, although adjustment reduced the significance of this difference. The amyloid plaques deposited within the vascular walls in CAA may possibly link to soluble tau proteins. Additionally, deposition of concomitant parenchymal amyloid plaques could explain the elevated soluble tau in CAA [31]. Our observations also agree with previous investigations and meta‐analyses, which found that CSF tau levels (including t‐tau and p‐tau) in CAA fall between the levels in AD and healthy controls [3, 32, 33, 34].

We did not observe significant differences in plasma Aβ40 or Aβ42 between our CAA and AD groups, even in the sensitivity analysis that excluded CAA cases with potential coexisting AD pathology. Previous research primarily focused on Aβ markers in CSF in CAA, rather than plasma [4, 5]. Lower CSF Aβ40 and Aβ42 levels were found in patients with CAA‐related hemorrhage compared to controls [32, 33, 35, 36, 37]. While low CSF Aβ40 may help to differentiate CAA from AD, the levels of Aβ42 in CSF were similar in these groups of patients [3]. These results support the hypothesis that Aβ40 peptide is deposited in vessels, leading to its depletion from CSF in advanced‐stage CAA. In contrast, inconsistent results have been reported for plasma Aβ levels when comparing CAA‐related ICH to controls [38]. Some studies suggest plasma Aβ levels may vary with the stage of CAA [5]. Unfortunately, our cohort size was insufficient to evaluate whether plasma Aβ40 or Aβ42 would differ between mild and severe CAA.

One interesting observation in this study is the significant correlation between plasma p‐tau 217 and the severity of CAA (as indicated by the MRI CAA‐SVD score), the lobar microbleed count, and cerebral amyloid burden on PET. We postulate that the intensification of CAA pathology leads to the release of p‐tau and the rupture of vessel walls as microbleeds. Similarly, our exploratory analysis revealed that increased plasma p‐tau 217 levels correlated with the severity of CAA. Moreover, higher p‐tau 217 levels were observed in mild CAA compared to the non‐amyloid DPA controls. This finding suggests that the release of p‐tau 217 into the bloodstream may occur early in the disease process in CAA. However, it remains unclear whether p‐tau 217 could serve as an ultra‐early marker for CAA‐laden vessels that are detectable before radiological damage, such as microbleeds, occurs. Further validation is required to confirm this hypothesis.

The optimal p‐tau 217 cutoff identified in this study had 64.4% sensitivity and 89.5% specificity to distinguish CAA from AD. Additional analysis in cognitively impaired patients recruited from memory clinics confirmed the diagnostic performance of p‐tau 217 (AUC 0.787, sensitivity 64.0%, specificity 89.5%). However, the ability of p‐tau 217 to differentiate concomitant CAA from AD, particularly in patients at risk of ARIA during immunotherapy, requires further evaluation [6]. Future work should focus on the identification of more specific plasma biomarkers that can detect coexisting CAA in AD.

In this study, the optimal cutoff for plasma p‐tau 217 had a high specificity (100%) to differentiate CAA from DPA, which indicates that individuals with DPA typically have p‐tau 217 levels below this threshold. Similarly, our additional analysis of ICH survivors (CAA‐ICH or DPA) validated the diagnostic accuracy of plasma p‐tau 217 (AUC 0.850, sensitivity 67.6%, specificity 95.5%). Overall, plasma p‐tau 217 may have diagnostic utility in different clinical settings: lower p‐tau 217 levels in memory clinic patients may help identify CAA and AD, while higher levels in survivors of symptomatic ICH may suggest underlying vascular amyloid pathology.

The strengths of this study include the integration of detailed amyloid and tau PET data, which enabled precise patient selection. Additionally, the inclusion of patients with DPA as an amyloid‐negative comparison group provided a clinically relevant comparison for the evaluation of plasma biomarkers in different subtypes of ICH. As the Boston criteria primarily diagnose CAA with strictly lobar hemorrhages, the incorporation of plasma biomarkers may address the diagnostic challenges related to mixed types of hemorrhage [1, 39].

Several limitations exist. First, the diagnoses were based on clinical presentation and imaging biomarkers rather than histopathological confirmation, which could lead to misclassification or mixed diagnoses. Concomitant Alzheimer's pathology in sporadic CAA, especially CAA presenting with cognitive impairment, could potentially confound our results. However, the main results remained unchanged in the sensitivity analysis that only included patients with CAA with negative tau PET scans. Second, there was substantial overlap in the p‐tau 217 levels across the groups. Future studies should focus on whether adding plasma p‐tau 217 enhances the diagnostic accuracy of the clinico‐radiological Boston criteria for CAA. Third, CSF was not analyzed in this study; thus, the correlations between the plasma and CSF levels of Aβ and tau biomarkers are unexplored. Fourth, we only assessed tau phosphorylated at the 217 site and did not explore whether other phosphorylation sites such as 181 or 231 may offer superior diagnostic potential for CAA. Fifth, no healthy controls were enrolled. Using symptomatic ICH in the DPA group as a non‐amyloid control may introduce bias toward more severe pathology and potentially reduce the discriminative power of the biomarkers evaluated. Lastly, further studies of plasma proteins related to other CAA pathophysiologies, such as white matter injury or neuroinflammation, are required to validate these plasma biomarkers.

## 5 Conclusion

Plasma p‐tau 217 may represent a non‐invasive biomarker, and it exhibited superior diagnostic performance over other plasma Aβ biomarkers for differentiating CAA from AD and CAA from DPA. Moreover, plasma p‐tau 217 correlated with the severity of the vascular lesions in CAA. Further investigation is warranted to delineate the mechanisms that lead to elevated p‐tau 217 in CAA.

## Author Contributions

**Pei‐Feng Hsieh:** conceptualization, methodology, data curation, writing – original draft. **Hsin‐Hsi Tsai:** conceptualization, methodology, software, data curation, investigation, validation, formal analysis, supervision, funding acquisition, project administration, resources, writing – review and editing. **Chia‐Ju Liu:** methodology, data curation, writing – review and editing. **Bo‐Ching Lee:** methodology, data curation, supervision, writing – review and editing. **Ya‐Chin Tsai:** data curation. **Ruoh‐Fang Yen:** conceptualization, supervision, writing – review and editing. **Jiann‐Shing Jeng:** data curation, investigation, writing – review and editing, project administration. **Li‐Kai Tsai:** conceptualization, methodology, data curation, investigation, supervision, project administration, writing – review and editing.

## Conflicts of Interest

The authors declare no conflicts of interest.

## Supporting information


Data S1.

## Data Availability

The data that support the current analysis are available upon reasonable request to the corresponding author.
