# Supplementary material for: Plasma Phosphorylated Tau 217 as a Discriminative Biomarker for Cerebral Amyloid Angiopathy
Source: Eur J Neurol. 2025 Feb 5;32(2):e70066. doi: 10.1111/ene.70066 (PMC11795418; doi:10.1111/ene.70066)
Supplement: Supplementary file 1 — Data S1. [file ENE-32-e70066-s001.docx]

**Supplementary Table 1. Comparison of core plasma biomarker levels between Tau PET-negative CAA and AD.**

|  | Tau (-) CAA (n=36) |  | AD  (n=19) | | p-value | Adjusted p^†^ | FDR-corrected p-value^‡^ |  |
| --- | --- | --- | --- | --- | --- | --- | --- | --- |
| Aβ 40, pg/mL | 234.38±96.36 |  | | 205.57±50.99 | 0.250 | 0.447 | 0.519 |  |
| Aβ 42, pg/mL | 10.93±5.60 |  | | 9.59±3.61 | 0.367 | 0.448 | 0.519 |  |
| p-tau 217, pg/mL | 0.67±0.93 |  | | 1.28±0.97 | *<0.001 | *0.002 | *0.005 |  |
| t-tau, pg/mL | 3.39±2.74 |  | | 3.21±1.72 | 0.684 | 0.454 | 0.519 |  |
| Aβ 42/40 | 0.05±0.01 |  | | 0.05±0.01 | 0.571 | 0.587 | 0.587 |  |
| p-tau217/t-tau | 0.23±0.19 |  | | 0.51±0.37 | *0.001 | *0.012 | *0.024 |  |
| p-tau217/Ab40 | 0.003±0.002 |  | | 0.006±0.003 | *<0.001 | *0.00038 | *0.003 |  |
| p-tau217/Ab42 | 0.060±0.045 |  | | 0.131±0.058 | *<0.001 | *0.001 | *0.004 |  |

Values are mean (± standard deviation). * *p*-value <0.05.

†Adjusted for age by quantile regression.

‡ Adjusted for age by quantile regression, and false discovery rate correction

Aβ: amyloid beta; AD: Alzheimer’s disease; CAA: Cerebral amyloid angiopathy; DPA: deep perforator arteriopathy; FDR: false discovery rate; p-tau: phosphorylated tau; t-tau: total tau.

**Supplementary Table 2. Comparison of core plasma biomarker levels between CAA-cognitive impairment (CI) and CAA-ICH.**

|  | All CAA  (*n*=59) | CAA-CI  (*n*=25) | CAA-ICH  (*n*=34) | *P* |
| --- | --- | --- | --- | --- |
| Aβ40, pg/mL | 232.05±87.60 | 236.49±77.00 | 228.79±95.65 | 0.334 |
| Aβ42, pg/mL | 10.77±5.07 | 10.60±4.23 | 10.89±5.67 | 0.830 |
| P-tau217 | 0.69±0.76 | 0.62±0.35 | 0.73±0.96 | 0.842 |
| Tau, pg/mL | 2.92±2.30 | 2.86±1.58 | 2.96±2.74 | 0.575 |
| Aβ42/40 | 0.05±0.01 | 0.05±0.01 | 0.05±0.01 | 0.818 |
| p-tau217/t-tau | 0.29±0.22 | 0.30±0.25 | 0.29±0.20 | 0.713 |
| p-tau217/Aβ40 | 0.003±0.002 | 0.003±0.002 | 0.003±0.002 | 0.713 |
| p-tau217/Aβ42 | 0.066±0.044 | 0.068±0.055 | 0.064±0.035 | 0.634 |

Values are mean (± standard deviation). * *p*-value <0.05.

Aβ: amyloid beta; CAA: Cerebral amyloid angiopathy; CI: cognitive impairment; ICH: Intracerebral hemorrhage; p-tau: phosphorylated tau; t-tau: total tau.

**Supplementary Table 3. Diagnostic performance of phosphorylated tau 217 in the memory clinic cohort and ICH cohort.**

|  | **Cutoff** | **Sensitivity** | **Specificity** | **AUC** |
| --- | --- | --- | --- | --- |
| **Memory cohort**  **(CAA vs. AD)** | <0.637  pg/mL | 0.640  (0.425-0.820) | 0.895  (0.668-0.987) | 0.787  (0.655-0.920) |
| **ICH cohort**  **(CAA vs. DPA)** | >0.448  pg/mL | 0.676  (0.495-0.826) | 0.955  (0.772-0.999) | 0.850  (0.752-0.948) |

Aβ: amyloid beta; AD: Alzheimer’s disease; AUC: area under the curve; CAA: cerebral amyloid angiopathy; DPA: deep perforator arteriopathy.
